# Predictors and Predictive Score of In-Hospital Mortality in Diabetic Ketoacidosis: A Retrospective Cohort Study

**DOI:** 10.3390/medicina60111833

**Published:** 2024-11-08

**Authors:** Neera Munsakul, Worapaka Manosroi, Supawan Buranapin

**Affiliations:** 1 Internal Medicine Department, Faculty of Medicine, Chiang Mai University, Chiang Mai 50200, Thailand; neeranam14@gmail.com; 2 Endocrine and Metabolism Unit, Internal Medicine Department, Faculty of Medicine, Chiang Mai University, Chiang Mai 50200, Thailand

**Keywords:** diabetic ketoacidosis, hyperglycemia crisis, mortality, predictors, predictive score

## Abstract

*Background and Objectives*: Diabetic ketoacidosis (DKA) is a critical complication of diabetes mellitus (DM). The primary objective of this study was to identify relevant clinical and biochemical predictors and create a predictive score for in-hospital DKA mortality. *Materials and Methods*: A 6-year retrospective cohort study of adult patients diagnosed with DKA and admitted to Chiang Mai University Hospital, a tertiary care center in Chiang Mai, Thailand, from 1 January 2015 to 31 December 2021, was conducted. Baseline clinical data and laboratory investigations were collected. The primary outcome was in-hospital mortality. Multivariable logistic regression analysis, clustered by type of diabetes, was performed to identify significant predictors. A predictive risk score was created using significant predictive factors identified by multivariable analysis. The results were presented as odds ratios (ORs) and 95% confidence intervals (CIs), with a significant *p*-value set at <0.05. *Results*: Ninety-three patients diagnosed with DKA were included in the study. Ten patients died during admission. Significant predictors for in-hospital mortality of DKA included age > 55 years (OR 7.8, *p* = 0.007), female gender (OR 3.5, *p* < 0.001), anion gap > 30 mEq/L (OR 2.6, *p* = 0.003), hemoglobin levels < 10 g/dL (OR 16.9, *p* < 0.001), and the presence of cardiovascular disease (OR 1.3, *p* = 0.046). The predictive risk score ranged from 1 to 14 for low risk, and 14.5–23.5 for high risk of in-hospital mortality. The predictive performance of the scoring system was 0.82 based on the area under the curve, with a sensitivity of 73.8% and specificity of 96.4%. *Conclusions*: Multiple clinical and biochemical factors, along with a predictive risk score, could assist in predicting in-hospital mortality of DKA and serve as a guide for physicians to identify patients at high risk. Nevertheless, as the predictive score was internally validated with data from a single institution, external validation in diverse healthcare settings with larger datasets or prospective cohorts is crucial to confirm the model’s generalizability and predictive accuracy.

## 1. Introduction

Diabetes mellitus (DM) is a major global public health concern. The worldwide incidence of DM and age-standardized incidence rates have shown a significant increase [1]. Approximately 90% of patients have type 2 DM, with the remaining cases encompassing other types of DM, including type 1 DM [2]. Diabetic ketoacidosis (DKA) is recognized as one of the most serious acute complications of DM. It is characterized by a triad of hyperglycemia, ketosis and high anion gap metabolic acidosis [3].

The incidence of DKA is as high as 56 per 1000 persons among adult patients with type 1 DM and 0.48 per 1000 persons among adult patients with type 2 DM [4,5]. Several epidemiological studies have reported a worldwide increase in hospitalizations for DKA among both type 1 and type 2 DM patients [6]. The overall mortality rate of DKA ranges from 0.2% to 2%, while for DKA overlapping with hyperosmolar hyperglycemia state (HHS), it is 8.3% [7]. In a previous prospective study, it was indicated that favorable outcomes in terms of in-hospital mortality from DKA were associated with male gender, decreased APACHE II score, and decreased serum phosphate levels [8]. Another study conducted in 2002 identified six predictors associated with in-hospital mortality, including severe co-existing disease, blood pH < 7.0, 50 or more units of insulin required in the first 12 h, serum glucose > 300 mg/dL after 12 h, depressed mental state, and presence of fever after 24 h. That study also developed a predictive model for in-hospital DKA mortality [9]. However, internal and external validation of the model were not performed in that study.

Given that the predictive model for in-hospital mortality among patients with DKA was established two decades ago without subsequent validation, this study aims to evaluate relevant clinical and laboratory parameters for predicting in-hospital mortality. Another objective is to develop and internally validate a predictive scoring system specifically tailored for in-hospital mortality in patients with DKA.

## 2. Materials and Methods

A 6-year retrospective cohort study of adult patients diagnosed with DKA and admitted to Chiang Mai University Hospital, a tertiary care center in Chiang Mai, Thailand, from 1 January 2015 to 31 December 2021, was conducted. Ethical approval for this study was waived by the Ethical Committee of the Faculty of Medicine, Chiang Mai University (Ethical number MED-2565-08797, date 21 January 2023). Informed consent was not required as this was a retrospective study. Patients aged over 18 who met the diagnostic criteria for DKA according to the American Diabetes Association upon admission were included [10]. Exclusion criteria included patients diagnosed with DKA after hospital admission, those for whom data retrieval for over 75% of the predefined factors was not possible, and pregnant individuals.

Clinical data and laboratory investigations obtained from electronic medical records were collected both prior to admission and during inpatient care. The clinical data included baseline demographic information, diabetes type, prior insulin therapy, type and dosage of oral diabetic agents, history of diabetes complications, comorbidities, precipitating factors for DKA, vital signs, Glasgow Coma Scale (GCS) scores, critical care predictive scores (APACHE II and SOFA score), DKA severity, admission location, and inpatient complications. Laboratory investigations comprised basic biochemical tests, baseline HbA1c, blood pH and serum ketone levels. The primary outcome of interest was in-hospital mortality.

### Statistical Analysis

The data were analyzed using STATA software version 17.0 (StataCorp, Lakeway, TX, USA). Continuous data are presented as mean ± standard deviation (SD). Categorical data are presented as the number and percentage. For inferential statistics, chi-squared or Fisher’s exact test were used for categorical data. Continuous data were analyzed using the independent t-test for normally distributed data or the Mann–Whitney U test for non-normally distributed data. Variables with missing data > 5% were dealt with using multiple imputation regression. Predictors of final outcomes and the predictive model were evaluated using multivariate stepwise logistic regression analysis clustered by the type of DM. The final model incorporated predictors selected based on their statistical significance in univariate analysis as well as the predictors that had clinical significance. Continuous variables were transformed to categorical variables in the final predictive model based on median values. Item scores were determined by transforming regression coefficients, with each factor’s coefficient divided by the smallest coefficient in the model and rounded to the nearest 0.5. These item scores were combined to derive a total score. Then, the total scores were categorized into two risk groups: low risk and high risk for DKA in-hospital mortality. The threshold for risk categorization was determined based on the level that yielded the highest sensitivity and specificity. The performance of the predictive model was assessed using the area under the receiver operating characteristic curve (AuROC). Internal validation was performed using the Bootstrap method. Statistical significance was set at a *p*-value < 0.05. A sample size of at least 95 patients was estimated to give 80% power at the 5% significance level (two-sided), with a hazard ratio of 2.5 for a specific predictor to predict in-hospital mortality.

## 3. Results

### 3.1. Baseline Demographic and Biochemical Investigations

Out of the 100 patients screened from medical records, 93 fulfilled the inclusion criteria (7 patients were diagnosed with DKA after hospital admission), with males comprising 50.5%. The average age of the patients was 52.3 ± 17.8 years. The majority of patients (79.6%) had type 2 DM, 18.3% had type 1 DM, and 2.1% had other types of DM. The mean length of hospital stay was 7.8 ± 6.2 days. Most participants did not have previous DM complications (76.3%). The most common precipitating factors were poor compliance with DM medications (21.5%) followed by infection (20.4%). The majority of the patients were admitted to the general ward (52.7%). The severity of DKA was mostly severe (52.7%), followed by moderate severity (21.5%). The mean HbA1c before admission was 9.5 ± 2.7%.

During admission, 10 patients (10.7%) died. Among these, patients admitted to the critical care unit, patients who had no prior insulin use, lower systolic blood pressure, lower GCS scores, higher APACHE II and SOFA scores, lower HbA1c levels before admission, lower hemoglobin levels, higher serum creatinine levels, and higher serum phosphate levels were associated with a higher mortality rate. There was no observed association between the severity of DKA and mortality rate. Baseline demographic data and biochemical investigations are presented in Table 1 and Table 2, respectively.

The primary complications of DKA recorded in this study were acute kidney injury, which was observed in 42 patients (45.2%), and infection, observed in 23 patients (24.7%) (Supplementary Table S1).

### 3.2. Predictors and Predictive Model of In-Hospital Mortality

**Figure 1:**
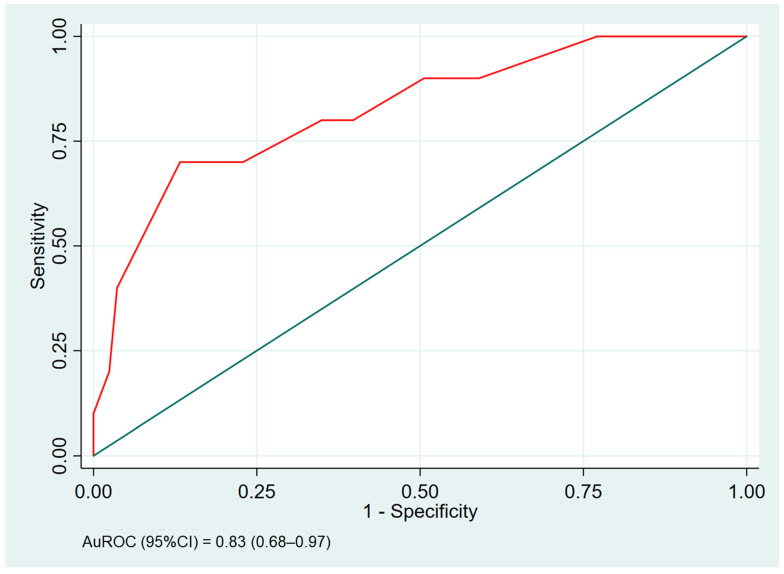
Area under the receiver operating characteristic (AuROC) of the predictive model of in-hospital death predicted by the predictive factors (red curved line) and a 50% chance of prediction (diagonal line).

The multivariate logistic regression analysis identified several predictive factors for in-hospital mortality among DKA patients. These included age > 55 years (OR 7.8, *p* = 0.007), female gender (OR 3.5, *p* < 0.001), anion gap > 30 mEq/L (OR 2.6, *p* = 0.003), hemoglobin levels < 10 g/dL (OR 16.9, *p* < 0.001), and the presence of cardiovascular disease (OR 1.3, *p* = 0.046). None of the other variables showed a significant association with the outcome. The receiver operating characteristic (ROC) curve for the predictive model incorporating all significant factors yielded an AuROC of 0.83 (0.68–0.97). Data are shown in Table 3 and Figure 1.

### 3.3. Predictive Scoring System of In-Hospital Mortality

A scoring system was developed to estimate the likelihood of in-hospital mortality, with item scores ranging from 1.0 to 9.0 and a total score of 23.5. This total score was divided into two categories: low risk for in-hospital mortality (scores of 1–14) and high risk for in-hospital mortality (scores of 14.5–23.5). This cut-off exhibited a sensitivity of 73.8%, specificity of 96.4% and positive likelihood ratio of 11.07. The scoring scheme is detailed in Table 4. The predictive performance of the scoring system, incorporating all five predictive factors, indicated an AuROC of 0.82 (95% CI 0.67–0.97), which was similar to the predictive ability of the model before conversion to the scoring system (AuROC 0.83, 95% CI 0.68–0.97). Furthermore, the model’s accuracy was validated through bootstrap internal validation, yielding a C-index of 0.93 (95% CI 0.82–0.97).

## 4. Discussion

This study identified five predictors of in-hospital mortality: age, sex, anion gap, hemoglobin levels, and the presence of cardiovascular disease. The proposed predictive score, which incorporated these five predictors and underwent internal validation, exhibited high predictive accuracy in predicting in-hospital mortality for patients with DKA. The integration of these predictors and the predictive score into routine clinical practice may help physicians identify high-risk DKA patients, enabling them to implement targeted interventions and allocate resources more efficiently. This, in turn, could lead to reduced mortality rates and enhanced quality of care for DKA.

The present study revealed a mortality rate of 10.7%. Comparatively, the overall global mortality rate of DKA was approximately 0.4–1.1% [11]. Previous studies across various ethnicities have shown mortality rate ranges from less than 1% to 44% [7,12,13,14]. The mortality rate observed in this study aligns closely with another study from Thailand, which shared the same resources and ethnicity as the present study, demonstrating an overall mortality rate of hyperglycemic crisis of 8.4% [7]. The higher mortality rate in the present study may be caused from differences in clinical settings and limitations in healthcare resources. This elevated mortality rate underscores the critical need for improvement in both pre-hospital and in-hospital care for DKA patients.

Multiple clinical and biochemical predictors have shown significant associations with in-hospital mortality in patients with DKA. Some of these predictors, such as age, hemoglobin levels, anion gap and underlying cardiovascular disease, have been previously identified in studies examining predictors of DKA outcomes [15,16,17]. In terms of advanced age, a previous study demonstrated a hazard ratio of 1.1 for 30-day mortality, whereas our study revealed an odds ratio of 7.8 for individuals aged over 55 years [15]. This disparity in age-related risk could be attributed to differences in study populations; the previous study had a lower mean age and a higher proportion of type 1 DM patients compared to our study. Younger patients with type 1 DM may exhibit lower mortality rates compared to elderly individuals, potentially influencing the observed variations in risk.

In a previous study, it was found that low hemoglobin levels were correlated with prolonged hospital stays; however, this study did not specify the threshold for defining low hemoglobin levels [16]. In contrast, our study determined that a hemoglobin level below 10 g/dL was associated with a 16.9-fold increase in in-hospital mortality of DKA. To our knowledge, there have been no studies demonstrating a link between low hemoglobin levels and in-hospital mortality in DKA. However, several studies in critical care settings have shown that anemia is associated with an increased risk of cardiac-related morbidity and mortality [18,19]. This increased mortality rate due to anemia may be attributed to a decrease in oxygen-carrying capacity, resulting in a mismatch in oxygen consumption within vital organs, especially organs that are highly dependent on oxygen such as the cerebrovascular and cardiovascular system [20]. Moreover, when oxygen levels are insufficient, cells switch from aerobic to anaerobic metabolism to produce energy. Anaerobic metabolism is less efficient and leads to the accumulation of lactic acid, causing metabolic acidosis. This can impair cellular function and contribute to organ dysfunction [21].

While no direct association between the anion gap and mortality rate in DKA was reported, one study did find that serum lactate levels ≥ 4 mmol/L were independent predictors of 5-day mortality in DKA patients, while our study found that an anion gap > 30 mEq/L was associated with increased in-hospital mortality in DKA [17]. Severe metabolic acidosis in DKA may serve as a marker for the severity of the condition, indicating the presence of organ dysfunction, impaired renal function, and systemic dysfunction. This observation suggests that patients with severe metabolic acidosis are at a higher risk of in-hospital mortality due to the underlying severity of DKA and associated complications. Additionally, metabolic acidosis can decrease myocardial contractility, worsen renal function, disrupt metabolic pathways, and impair cellular metabolism, all of which may contribute to a higher mortality rate [22].

In a prior study, individuals with multiple severe co-existing diseases, including prior myocardial infarction and congestive heart failure, were found to have a 16.3-fold increased risk of mortality associated with DKA [9]. In contrast, our study revealed a lower increase in in-hospital mortality for DKA patients with underlying cardiovascular disease, specifically a 1.4-fold increase. This substantial difference in the risk of DKA mortality could be attributed to variations in the definition of co-existing diseases, as the prior study included other co-morbidities apart from cardiovascular disease. Notably, our study focused solely on underlying cardiovascular disease, which included underlying myocardial infarction, heart failure and revascularization.

To the best of our knowledge, there was no reported association between gender and mortality in DKA. The present study revealed that females faced a 3.5-fold higher risk of in-hospital mortality due to DKA. This finding aligns with the results of a meta-analysis on sex differences in severity and mortality among critically ill patients, which indicated that females tended to have higher illness severity scores upon admission and a higher risk-adjusted mortality rate than men at discharge and at 1-year post-discharge [23]. There is also evidence suggesting that estrogen and progesterone can affect immune function [24]. Other predictors, such as the insulin unit used in the first 12 h, serum glucose levels, depressed mental status, fever after 24 h, APACHE II score and serum sodium during admission, have been reported as predictors of DKA mortality in previous studies. However, these factors were not identified as significant predictors in the present study [7,8,9].

The present study developed a novel scoring system to predict in-hospital mortality for adult DKA patients, which represents the latest scoring system since the previous system was introduced in 2002 [9]. The prior predictive score may not be as relevant in current clinical practice due to changes in medical resources and updates in clinical guidelines. This new scoring system integrated both simple and commonly used biochemical and laboratory data collected at the baseline of patient admission. Additionally, internal validation was conducted, demonstrating high accuracy in predicting DKA mortality. The study’s sample size provided sufficient power for analysis. The observed correlation between predictors and outcomes was unlikely to be coincidental, as all predictors were consistent and could be explained with the underlying pathophysiology of DKA.

There are some limitations in this study. Firstly, the predictive score was internally validated using data from a single institution, performing external validation in other healthcare settings or larger datasets essential to confirm the generalizability of the model and its predictive ability. Secondly, being a retrospective study, some missing data might have occurred. Additionally, the small sample size must be taken into consideration. Serum ketone levels could not be retrieved from the database because, at our institution, they were measured using a qualitative method rather than a quantitative one.

## 5. Conclusions

DKA is one of the most serious complications of DM. Reducing the mortality rate associated with DKA remains a significant challenge. This study has identified multiple predictive factors of in-hospital mortality of DKA at the baseline of patient admission. Integrating these predictors and the predictive score into routine clinical practice may assist physicians in identifying high-risk DKA patients. Future external validation using a larger cohort and an exploration of how the predictive score could be integrated into various clinical workflows would enhance the practical impact of this predictive risk score.

## Figures and Tables

**Table 1:** Baseline demographic data (*n* = 93).

Characteristics	Total	Death in Hospital (*n* = 10)	Discharged Alive (*n* = 83)	*p*-Value
Male, *n* (%)	47 (50.5)	4 (40.0)	43 (51.8)	0.480
Age, year (mean ± SD)	52.3 ± 17.8	58.9 ± 18.7	51.5 ± 17.6	0.218
BMI, kg/m^2^ (mean ± SD)	22.9 ± 4.9	21.1 ± 5.1	23.1 ± 4.9	0.291
Smoking, *n* (%)	18 (19.4)	1 (10.0)	17 (20.5)	0.428
Type of DM				0.878
Type 1 DM, *n* (%)	17 (18.3)	2 (20.0)	15 (18.1)	-
Type 2 DM, *n* (%)	74 (79.6)	8 (80.0)	66 (79.5)	-
Other types of DM, *n* (%)	2 (2.1)	0 (0)	2 (2.4)	-
Prior insulin use, *n* (%)	58 (62.4)	3 (30.0)	55 (66.3)	0.025
TDD of basal insulin, unit (mean ± SD)	17.9 ± 19.6	7.8 ± 16.6	19.2 ± 19.7	0.083
Oral diabetes agents				0.568
Metformin, *n* (%)	43 (46.2)	4 (40.0)	39 (47)	-
Sulfonylurea, *n* (%)	23 (24.7)	3 (30.0)	20 (24.1)	-
SGLT2i, *n* (%)	8 (8.6)	0 (0)	8 (9.6)	-
DPP4i, *n* (%)	12 (12.9)	0 (0)	12 (14.5)	-
Pioglitazone, *n* (%)	15 (16.1)	1 (10)	14 (16.9)	-
GLP1 agonist, *n* (%)	1 (1.1)	0 (0)	1 (1.2)	-
Previous DM complication				0.772
None, *n* (%)	71 (76.3)	9 (90.0)	62 (74.4)	-
Diabetic retinopathy, *n* (%)	4 (4.3)	1 (10.0)	3 (3.6)	-
Diabetic nephropathy, *n* (%)	6 (6.5)	0 (0)	6 (7.2)	-
Diabetic foot, *n* (%)	0 (0)	0 (0)	0 (0)	-
Previous DKA, *n* (%)	7 (7.5)	0 (0)	7 (8.4)	-
>1 complication, *n* (%)	5 (5.4)	0 (0)	5 (6)	-
Other underlying disease				
Cardiovascular disease *, *n* (%)	5 (5.4)	1 (10.0)	4 (4.8)	0.493
Stroke, *n* (%)	1 (1.1)	0 (0)	1 (1.2)	0.727
CKD, *n* (%)	14 (15.1)	2 (20.0)	12 (14.5)	0.643
OSA, *n* (%)	1 (1.1)	0 (0)	1 (1.2)	0.727
Hypertension, *n* (%)	50 (53.8)	6 (60.0)	44 (53)	0.675
Dyslipidemia, *n* (%)	40 (43)	3 (30.0)	37 (44.6)	0.379
Admission Data				
Place of admission				0.028
General ward, *n* (%)	49 (52.7)	2 (20.0)	47 (56.6)	-
Critical care unit, *n* (%)	44 (47.3)	8 (80.0)	36 (43.4)	-
Precipitating factors				<0.001
Infection, *n* (%)	19 (20.4)	0 (0)	19 (22.9)	-
Poor compliance with medications, *n* (%)	20 (21.5)	1 (10.0)	19 (22.9)	-
Combination of infection and poor compliance, *n* (%)	48 (51.6)	6 (60.0)	42 (50.6)	-
Others, *n* (%)	6 (6.5)	3 (30.0)	3 (3.6)	-
SBP, mmHg (mean ± SD)	127.2 ± 25.6	104.5 ± 20.4	129.9 ± 24.8	0.003
DBP, mmHg (mean ± SD)	74.9 ± 19	65.5 ± 23.1	76 ± 18.3	0.099
GCS (mean ± SD)	12.9 ± 3.6	7 ± 3.9	13.7 ± 2.8	<0.001
APACHE II (mean ± SD)	15.3 ± 8	27.8 ± 7.1	13.8 ± 6.7	<0.001
SOFA score (mean ± SD)	2.7 ± 2.9	8.1 ± 0.7	2.1 ± 2.4	<0.001
Severity of DKA				0.873
Mild, *n* (%)	24 (25.8)	2 (20.0)	22 (26.5)	-
Moderate, *n* (%)	20 (21.5)	2 (20.0)	18 (21.7)	-
Severe, *n* (%)	49 (52.7)	6 (60.0)	43 (51.8)	-

BMI: body mass index, DM: diabetes mellitus, TDD: total daily dose, SGLT2i: sodium glucose cotransporter-2 inhibitors, DPP4i: dipeptidyl peptidase-4 inhibitors, GLP1: glucagon-like peptide-1, CKD: chronic kidney disease, OSA: obstructive sleep apnea, SBP: systolic blood pressure, DBP: diastolic blood pressure, DKA: diabetic ketoacidosis, GCS: Glasgow Coma Scale, APACHE II: Acute Physiology and Chronic Health Evaluation II, SOFA; Sequential Organ Failure Assessment. * Myocardial infarction, heart failure, revascularization.

**Table 2:** Baseline biochemical investigations (*n* = 93).

Biochemical Profiles	Total	Death in Hospital (*n* = 10)	Discharged Alive (*n* = 83)	*p*-Value
HbA1C before admission, % (mean ± SD)	9.5 ± 2.7	6.9 ± 2.6	9.8 ± 2.6	0.012
HbA1C during admission, % (mean ± SD)	10.9 ± 2.9	9.9 ± 1.4	11.1 ± 2.9	0.219
Hemoglobin, g/dL (mean ± SD)	13.2 ± 2.7	11.3 ± 3.8	13.5 ± 2.4	0.012
White blood cell, cell/cu.mm. (mean ± SD)	16,345.6 ± 15,400.9	24,846 ± 20,973.6	15,321.5 ± 14,420.9	0.064
Platelet count, cell/cu.mm. (mean ± SD)	293,863.4 ± 117,201	270,800 ± 141,340	296,642.2 ± 114,655	0.513
Plasma glucose, mg/dL (mean ± SD)	599.3 ± 267.9	695.8 ± 265.6	587.7 ± 267.4	0.230
Blood pH (mean ± SD)	7.2 ± 0.2	7.1 ± 0.1	7.2 ± 0.2	0.409
Serum creatinine, mg/dL (mean ± SD)	1.8 ± 1.9	3.2 ± 2.8	1.6 ± 1.7	0.012
Serum sodium, mEq/L (mean ± SD)	129.3 ± 7.1	129 ± 6.7	129.3 ± 7.2	0.904
Serum corrected sodium, mEq/L (mean ± SD)	140.8 ± 7.9	142.9 ± 10.5	140.5 ± 7.6	0.359
Serum potassium, mEq/L (mean ± SD)	5.1 ± 0.9	5.2 ± 0.7	5.1 ± 1	0.640
Serum HCO3, mEq/L (mean ± SD)	9.9 ± 4.7	9.9 ± 4.4	9.9 ± 4.7	0.984
Anion gap, mEq/L (mean ± SD)	30.8 ± 7.2	32 ± 5.2	30.7 ± 7.4	0.587
Serum phosphate, mg/dL (mean ± SD)	5.3 ± 2.8	7.4 ± 2.3	5.1 ± 2.8	0.014
Serum total bilirubin, mg/dL (mean ± SD)	0.8 ± 1.2	0.9 ± 0.9	0.7 ± 1.2	0.601
SGOT, U/L (mean ± SD)	50 ± 152.5	79.8 ± 81.1	46.2 ± 159.2	0.515
Serum albumin, g/dL (mean ± SD)	4.7 ± 7	3.6 ± 0.7	4.8 ± 7.5	0.628

Serum corrected sodium = measured sodium + [1.6 (glucose − 100)/100].

**Table 3:** Predictors of in-hospital death of DKA analyzed by multivariate logistic regression analysis clustered by type of diabetes.

Predictors	OR	95% CI	*p*-Value
Age > 55 years	7.8	1.7–35.2	0.007
Female	3.5	2.8–4.3	<0.001
Anion gap > 30 mEq/L	2.6	1.4–4.9	0.003
Hemoglobin < 10 g/dL	16.9	8.5–33.8	<0.001
Had cardiovascular disease	1.4	1.1–1.6	0.005

OR: Odds ratio.

**Table 4:** Predictive scoring system of in-hospital mortality of DKA.

Predictor	β-Coefficient	Transformed Coefficient	Item Score
Age > 55 years	2.05	6.40	
Yes			6.5
No			0
Sex	1.25	3.90	
Female			4
Male			0
Anion gap > 30 mEq/L	0.97	3.03	
Yes			3
No			0
Hemoglobin < 10 g/dL	2.83	8.84	
Yes			9
No			0
Had cardiovascular disease	0.32	1	
Yes			1
No			0
Total score			23.5

## Data Availability

The data that support the findings of this study are available upon request from the authors.

## Supplementary Materials

The following supporting information can be downloaded at https://www.mdpi.com/article/10.3390/medicina60111833/s1: Table S1: Complications during admission.
