# Supplementary material for: Predictors and Predictive Score of In-Hospital Mortality in Diabetic Ketoacidosis: A Retrospective Cohort Study
Source: Medicina (Kaunas). 2024 Nov 8;60(11):1833. doi: 10.3390/medicina60111833 (PMC11596054; doi:10.3390/medicina60111833)
Supplement: Supplementary file 1 [file medicina-60-01833-s001.zip › medicina-3257074-supplementary.pdf]

Supplementary Table

Table S1. Complications during admission

**Other complication**	**Total**	**Death in hospital**	**Discharged alive**	**p-value**
Acute kidney injury, n (%)	42 (45.2)	6 (60)	36 (43.4)	0.318
Acute heart failure, n (%)	6 (6.5)	3 (30)	3 (3.6)	0.001
Infection, n (%)	23 (24.7)	5 (50)	18 (21.7)	0.050
Septic shock, n (%)	12 (12.9)	7 (70)	5 (6)	<0.001
Inotropic drug use, n (%)	16 (17.2)	9 (90)	7 (8.4)	<0.001
Others, n (%)	24 (25.8)	5 (50)	19 (22.9)	0.064
